# Supplementary material for: Identification of Intron Retention in the Slc16a3 Gene Transcript Encoding the Transporter MCT4 in the Brain of Aged and Alzheimer-Disease Model (APPswePS1dE9) Mice
Source: Genes (Basel). 2023 Oct 17;14(10):1949. doi: 10.3390/genes14101949 (PMC10606527; doi:10.3390/genes14101949)
Supplement: Supplementary file 1 [file genes-14-01949-s001.zip › genes-2651561-supplementary.pdf]

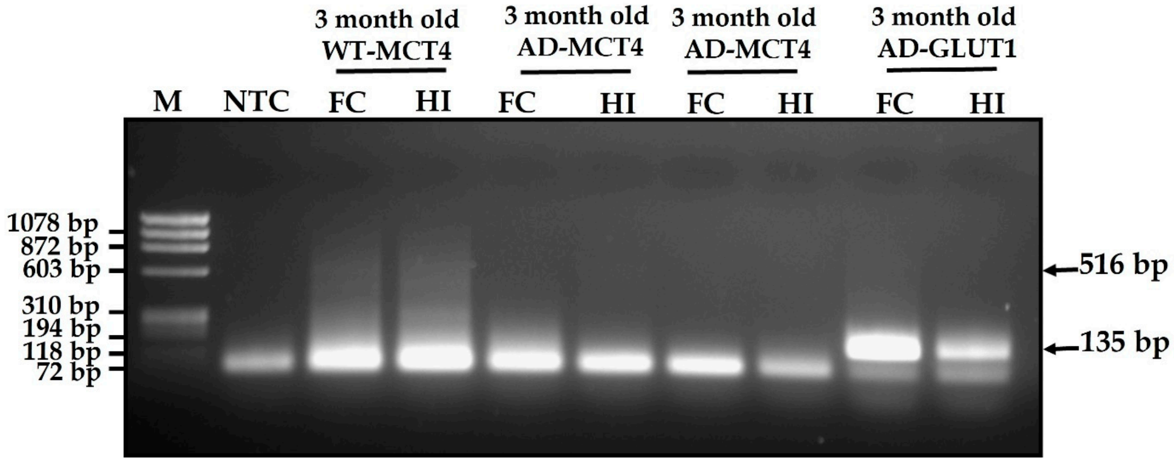

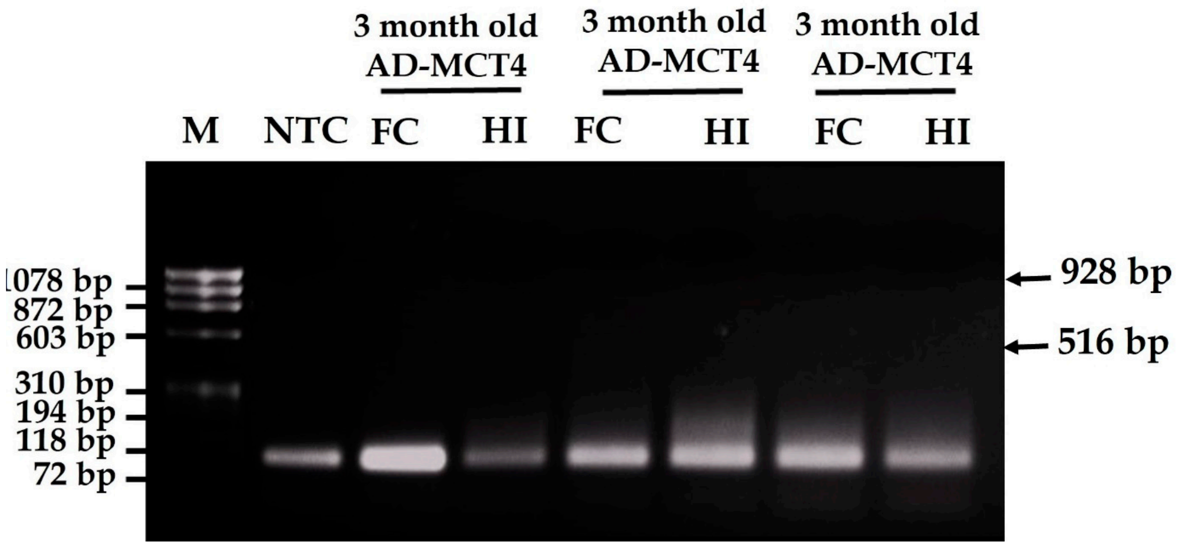

Figure S1. RT-PCR for the *Slc16a3* gene in the frontal cortex (FC) and hippocampus (HI) of 3month-old Alzheimer (AD) mice. As control, RT-sqPCR for the *Slc2a1* gene (coding for GLUT1) was performed and exhibited the expected 135 bp fragment.
